# Design, Synthesis, and In Vitro Evaluation of Novel Histone H3 Peptide-Based LSD1 Inactivators Incorporating α,α-Disubstituted Amino Acids with γ-Turn-Inducing Structures

**DOI:** 10.3390/molecules23051099

**Published:** 2018-05-06

**Authors:** Yosuke Ota, Taeko Kakizawa, Yukihiro Itoh, Takayoshi Suzuki

**Affiliations:** 1 Department of Chemistry, Graduate School of Medical Science, Kyoto Prefectural University of Medicine, 1-5 Shimogamohangi-Cho, Sakyo-Ku, Kyoto 606-0823, Japan; itohy@koto.kpu-m.ac.jp; 2 Department of Chemistry and Biochemistry, School of Advanced Science and Engineering, Waseda University, Shinjuku, Tokyo 169-8555, Japan; kg100683@kanto-gakuin.ac.jp; 3 Core Research for Evolutional Science and Technology (CREST), Japan Science and Technology Agency (JST), 4-1-8 Honcho, Kawaguchi, Saitama 332-0012, Japan

**Keywords:** epigenetics, histone, lysine-specific demethylase 1 (LSD1), inhibitor, γ-turn structure

## Abstract

Lysine-specific demethylase 1 (LSD1) mainly removes methyl groups of mono- or di-methylated lysine residues at the fourth position of histone H3 to epigenetically regulate the expression of genes associated with several diseases, such as cancer. Therefore, LSD1 inactivators are expected to be used as therapeutic agents. In this study, to identify novel peptide-based LSD1 inactivators, we focused on the X-ray structure of LSD1 complexed with a H3 peptide-based suicide substrate. It has been proposed that a methylated histone substrate forms three consecutive γ-turn structures in the active pocket of LSD1. Based on this, we designed and synthesized novel histone H3 peptide-based LSD1 inactivators **2a**–**c** by incorporating various α,α-disubstituted amino acids with γ-turn-inducing structures. Among synthetic peptides **2a**–**c**, peptide **2b** incorporating two 1-aminocyclohexanecarboxylic acids at both sides of a lysine residue bearing a *trans*-2-phenylcyclopropylamine (PCPA) moiety, which is a pharmacophore for LSD1 inactivation, was the most potent and selective LSD1 inactivator. These findings are useful for the further development of histone H3 peptide-based LSD1 inactivators.

## 1. Introduction

The methylation of lysine residues on histones is involved in the epigenetic control of gene expression associated with not only many cellular events but also several diseases [1]. Histone methylation and demethylation reactions are catalyzed by a series of histone lysine methyltransferases (HKMTs) and histone lysine demethylases (KDMs), respectively [1]. Thus, the methylation is reversibly regulated by both HKMTs and KDMs.

Lysine-specific demethylase 1 (LSD1), which is one of the KDMs and a flavin adenine dinucleotide (FAD)-dependent oxidase, catalytically removes the methyl groups of mono- or di-methylated lysine residues at the fourth position of histone H3 (H3K4me1/me2) [2]. In prostate cancer cells, LSD1 acquires H3K9me1/me2 demethylation activity by interacting with an androgen receptor [3]. In addition, LSD1 demethylates methyl groups of mono- or di-methylated lysine residues of non-histone proteins (e.g., p53, DNMT, and E2F1) to regulate their activities and functions [4,5,6]. LSD1 forms a co-repression complex with CoREST, recruiting other transcription factors, such as HDAC1, Gfi-1b, TLX, KHMTs, and other KDMs [7,8,9,10,11], to epigenetically regulate the expression of the genes associated with several diseases, such as cancer, globin disorders, and viral infections [12,13,14]. Therefore, LSD1 is interesting as a molecular target for the therapy of diseases. To date, a number of LSD1 inactivators have been developed [15,16,17] and some of them are being evaluated in clinical trials for cancer [18,19].

**Figure 1:**
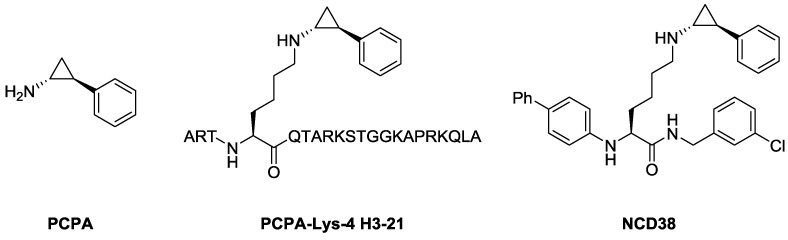
Structures of *trans*-2-phenylcyclopropylamine (PCPA) and its analogs as LSD1 inactivators.

We have reported several peptide- or small molecule-based LSD1 inactivators so far [20,21,22,23,24,25,26,27,28,29]. For instance, PCPA-Lys-4 H3-21, a peptide-based LSD1 inactivator [22] (Figure 1), drops off *trans*-2-phenylcyclopropylamine (PCPA), a pharmacophore for LSD1 inactivation [30,31], in the active pocket of LSD1 to inactivate LSD1 potently and selectively. Further, we identified NCD38, a potent and selective small molecule LSD1 inhibitor [22] (Figure 1) that showed sufficient bioactivity in in vivo studies [32,33]. Following these findings, we considered that it is possible to improve the LSD1 inhibitory activities of peptide- or small molecule-based LSD1 inactivators. In particular, a novel design of peptide-based LSD1 inactivators could lead to high specificity toward LSD1 and their off-target effects could be limited as compared with small molecule inhibitors. Thus, peptide-based LSD1 inactivators could exhibit high therapeutic potential toward LSD1-associated diseases [12,13,14].

**Figure 2:**
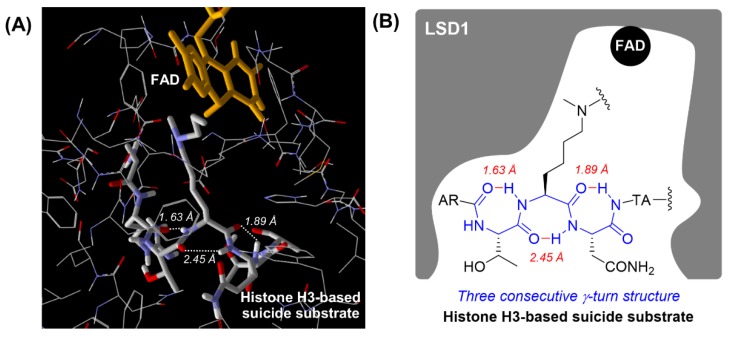
Secondary structure of a histone H3-based suicide substrate in the active pocket of LSD1 (PDB: 2UXN). (**A**) View of the secondary structure of the histone H3-based suicide substrate in the active pocket of LSD1. Intramolecular hydrogen bonds are indicated by the white dotted line and their lengths are shown in italics. (**B**) Schematic diagram of the secondary structure of the histone H3-based suicide substrate in the active pocket of LSD1. Intramolecular hydrogen bonds are indicated by the red line and their lengths are shown in italics.

In 2007, Cole, Yu, and co-workers reported a co-crystal structure of LSD1 with a histone H3-based suicide substrate incorporating *N*-methylpropargylamine, and revealed that the substrate reacted with FAD to form three consecutive γ-turn structures in the active pocket of LSD1 (Figure 2) [34]. The unique secondary structures could contribute to the substrate specificity toward LSD1 [34]. Based on that report, we envisioned that the LSD1 inhibitory activities of peptide-based inactivators would be improved by regulating their secondary structures. Thus, peptides that easily form three consecutive γ-turn structures should exhibit strong LSD1 inhibitory activity and high selectivity. Herein we report novel histone H3 peptide-based LSD1 inactivators incorporating α,α-disubstituted amino acids that work as γ-turn inducers.

## 2. Results

### 2.1. Design

**Figure 3:**
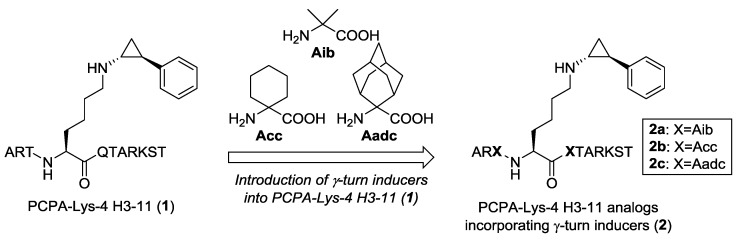
Structure of PCPA-Lys 4 H3-11 (**1**) (**left**). Design of novel histone H3 peptide-based LSD1 inactivators **2a**–**c** incorporating α,α-disubstituted amino acids that work as γ-turn inducers (**right**).

Previously, it was reported that α,α-disubstituted amino acids predominantly induced the formation of γ-turn structures [35]. Thus, we hypothesized that the introduction of α,α-disubstituted amino acids into the sequence of a histone H3-based peptide would stabilize the formation of three consecutive γ-turn structures in the active pocket of LSD1. To verify the hypothesis, we selected LSD1 inactivator **1** as the model and reference peptide (Figure 3), because peptide **1** has both a concise sequence and appropriate LSD1 inhibitory activity [28]. We designed histone H3 peptide-based LSD1 inactivators **2a**–**c** by incorporating two of the following α,α-disubstituted amino acids: 2-aminoisobutyric acid (Aib) (**2a**), 1-aminocyclohexanecarboxylic acid (Acc) (**2b**), and 2-aminoadamantane-2-carboxylic acid (Aadc) (**2c**), one on each side of the lysine residue bearing a PCPA moiety (Figure 3).

### 2.2. Synthesis

**Scheme 1:**
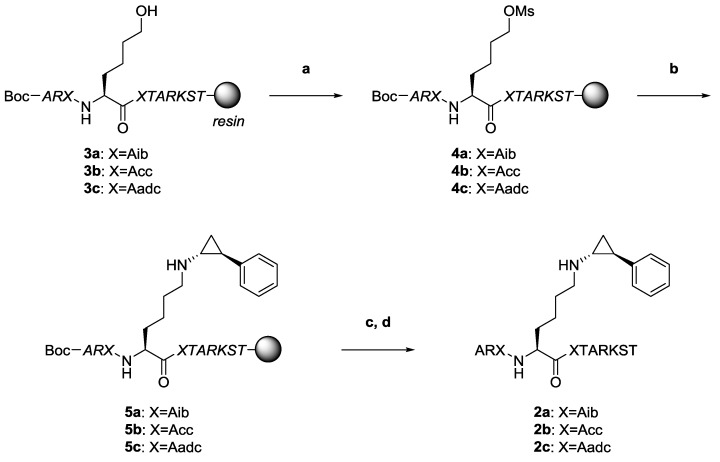
Preparation of novel histone H3 peptide-based LSD1 inactivators **2a**–**c** with γ-turn inducers by Fmoc-based SPPS (Boc-Ala-OH was used as the *N*-terminal amino acid). Reagents and conditions: (**a**) MsCl, Et_3_N in THF; (**b**) PCPA, Et_3_N in H_2_O:CH_3_CN solution; (**c**) TFA, thioanisole, H_2_O; and (**d**) preparative HPLC. Peptide chains shown in italics are protected.

The synthesis of H3-11 peptide analogs **2** is shown in Scheme 1. Peptides **2** were synthesized by Fmoc-based solid-phase peptide synthesis (SPPS) according to modified previous procedures for histone H3-based peptides [22,25,28]. Initially, we prepared peptides **3** incorporating the α,α-disubstituted amino acids by Fmoc-based SPPS. Next, peptides **3** were converted into mesylates **4** with mesyl chloride (MsCl), and then the mesyl groups of **4** were substituted with PCPA to obtain desired precursors **5**. Precursors **5** were cleaved from the resin and purified by high-performance liquid chromatography (HPLC) followed by lyophilization and identification by matrix-assisted laser desorption/ionization time-of-flight mass spectrometry (MALDI-TOF MS) to obtain the desired peptides **2** as a white powder.

### 2.3. In Vitro Evaluation of LSD1 Inhibitory Activity

We assayed for the LSD1 inhibitory activities of novel peptides **2a**–**c**. The assay was performed with an LSD1 Fluorometric Drug Discovery Kit (Enzo Life Sciences, New York, NY, USA, BML-AK544-0001). The results are summarized in Table 1. Control peptide **1** inactivated LSD1 potently compared to PCPA. As expected, the LSD1 inhibitory activities of novel peptides **2a**–**c** were higher than that of **1**. In particular, peptide **2b** was the most potent LSD1 inactivator among the peptides tested in this study. Next, we examined the time-dependency of LSD1 inhibition by peptides **2a**–**c**. As shown in Supplementary Figure S1, peptide **2a**–**c** inhibited LSD1 enzyme activities in a time-dependent manner, which indicates that peptides **2a**–**c** inactivate LSD1 irreversibly. We also assayed for the inhibitory activities of monoamine oxidases (MAOs), which are FAD-dependent oxidases associated with neurotransmitter metabolism [36], to investigate the selectivity toward LSD1. For this investigation, we used a MAO-Glo^TM^ Assay System (Promega, Madison, WI, USA, V1401). As shown in Table 1, all peptides did not inactivate MAO-A or MAO-B at the concentration of 10 µM, whereas PCPA, the positive control, inactivated both MAO-A and MAO-B. Furthermore, we tested the inhibitory activities of peptides **2a**–**c** for KDM4C, an isoform of α-ketoglutarate/Fe(II)-dependent KDMs [37]. As a result, peptides **2a**–**c** hardly inhibited KDM4C activities at a concentration of 10 μM (Supplementary Figure S2). Thus, the novel peptides **2a**–**c** were found to be irreversible LSD1-selective inactivators.

## 3. Discussion

In this work, we designed and synthesized γ-turn-inducing novel peptides **2a**–**c** incorporating α,α-disubstituted amino acids and evaluated them in an in vitro LSD1 inhibitory assay. We revealed that peptides **2a**–**c** were potent and selective LSD1 inactivators compared to reference peptide **1**. In particular, peptide **2b** incorporating one Acc each on both sides of the lysine residue bearing the PCPA moiety showed the most potent LSD1 inhibitory activity and the highest LSD1 selectivity (>170-fold).

**Figure 4:**
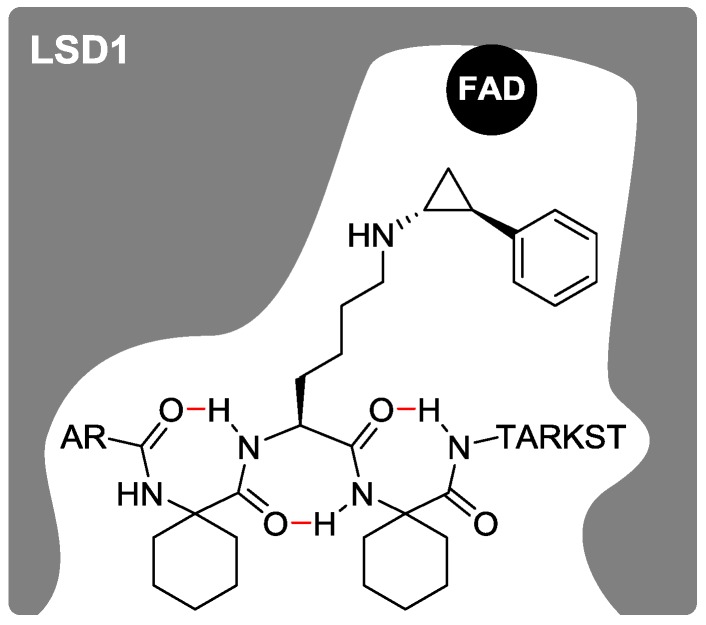
A proposed secondary structure of histone H3 peptide-based LSD1 inactivator **2b** in the active pocket of LSD1 is shown in a schematic diagram. Intramolecular hydrogen bonds are indicated in red.

Based on these results and our previous findings [22,25,28], we consider that peptides **2a**–**c** incorporating α,α-disubstituted amino acids as γ-turn inducers, particularly **2b**, form three consecutive γ-turn structures in the active pocket of LSD1 to be highly recognized by LSD1 and thereby strongly inactivating LSD1 (Figure 4). Indeed, our docking study using a short model peptide based on **2b** suggested that it forms three consecutive γ-turn structures and binds to the active pocket of LSD1 (Supplementary Figure S3). Although we have not obtained any experimental results regarding the secondary structures of the peptides incorporating α,α-disubstituted amino acids in the active pocket of LSD1, structural investigation using various approaches, such as NMR analysis and computational chemistry, is underway.

The structures of peptides are often a source of problems, such as low membrane permeability or low tolerance to proteases, but those problems could be solved by introducing drug delivery systems [38]. Meanwhile, it was reported that more hydrophobic histone H3-based cyclic peptides penetrate the cell membrane to exert an antiproliferative effect on cancer cells by inhibiting LSD1 in the cells, but less hydrophobic non-cyclic ones do not [39,40]. Recently, it was also reported that a more hydrophobic H3-based cyclic peptide stabilized by a disulfide bridge penetrates the cell membrane and inhibits LSD1, thereby inducing histone methylation, whereas a less hydrophobic non-cyclic one does not [41]. Thus, we speculate that our peptides incorporating α,α-disubstituted amino acids might passively penetrate the cell membrane to regulate LSD1 cellular function, because the peptides have hydrophobic properties and secondary structures stabilized by γ-turn inducers. In addition, it was reported that the introduction of unnatural amino acids into the original peptide sequence could improve the tolerance to protease [39,42]. Thus, we also expect that our peptides would exhibit protease tolerance. In the future, we are going to test the cellular activities of our peptides.

## 4. Materials and Methods

### 4.1. General Methods

All reagents were purchased and used as received. Fmoc-Aib-OH and Fmoc-Acc-OH were purchased from Watanabe Chemical Industries (Hiroshima, Japan). Fmoc-Aadc-OH was prepared from H-Aadc-OH (Ark Pharm, Arlington Heights, IL, USA) via a standard method for Fmoc introduction [43]. Obtained peptides were purified by preparative HPLC and the purity was estimated by analytical HPLC. Preparative HPLC was performed with a pump system of JASCO PU-2028 Plus (JASCO , Tokyo, Japan) and a Cosmosil 5C_18_-AR-II column (20 × 250 mm, Nacalai Tesque, Kyoto, Japan) using a linear gradient of 0.1% trifluoroacetic acid (TFA) in CH_3_CN and 0.1% aqueous TFA at the flow rate of 5.0 mL min^−1^, and detection was at 220 nm. Analytical HPLC was carried out with a pump system of JASCO PU-2028 Plus and a Cosmosil 5C_18_-AR-II column (4.6 × 250 mm, Nacalai Tesque) using a linear gradient of 0.1% TFA in CH_3_CN and 0.1% TFA in H_2_O (1.0 mL min^−1^, 220 nm). All peptides were characterized by MALDI-TOF MS using a Bruker Autoflex (Bruker, Billerica, MA, USA) with α-cyano-4-hydroxycinnamic acid (CHCA) as the matrix.

### 4.2. Synthesis of Peptides with γ-Turn Inducers

PCPA-Lys-4 peptides were synthesized using a modified strategy based on the published method for PCPA-Lys-4 H3-21 [22,25,28]. The synthesis of the protected mesyl-Lys-4 peptide sequence was accomplished on the resin based on standard Fmoc-based SPPS according to literature [44,45,46] and Boc-Ala-OH was used as the *N*-terminal amino acid. The resin was treated with 5 equivalents of *trans*-2-phenylcyclopropylamine hydrochloride and 10 equivalents of Et_3_N in 1:1 solution of H_2_O/CH_3_CN for 3 days at room temperature. Deprotection and cleavage from the resin were performed with TFA/thioanisole/H_2_O (85:10:5) solution. The desired peptide was collected by preparative RP-HPLC and lyophilized to give a white powder. MALDI-TOF MS (*m*/*z*): Calcd. for PCPA-Lys-4 H3-11 (**1**) ([M + H]^+^): 1364.529. Found: 1364.322. Calcd. for Aib peptide (**2a**) ([M + H]^+^): 1305.572. Found: 1305.155. Calcd. for Acc peptide (**2b**) ([M + H]^+^): 1385.702. Found: 1385.293. Calcd. for Aadc peptide (**2c**) ([M + H]^+^): 1489.854. Found: 1489.370.

### 4.3. Assay for LSD1 Inhibitory Activity

Assays for LSD1 inhibitory activity were performed using an LSD1 Fluorometric Drug Discovery Kit (Enzo Life Sciences, BML-AK544-0001). Inhibitors were pre-incubated with LSD1 (0.5 µg/well; room temperature; 15 min) before reactions were initiated by the addition of H3K4me2 peptide (2.3 µg/well, 20 µM) into all wells except the blank. After incubation (room temperature; 30 min), CeLLestial^TM^ Red and HRP in the assay buffer were added and the incubation was prolonged (room temperature; 5 min). The fluorescence of the wells was measured on a 2030 ARVO^TM^ X3 Multilabel Reader (PerkinElmer, Waltham, MA, USA, excitation: 540 nm; detection: 590 nm) and % inhibition was calculated from the fluorescence readings of inhibited wells relative to those of control wells. The concentration of test compounds that resulted in 50% inhibition was determined by plotting log [Inh] against the logit function of the % inhibition. IC_50_ values were determined by regression analysis of the concentration/inhibition data.

### 4.4. Assay for MAO Inhibitory Activity

Assays for MAO inhibitory activity were carried out according to the supplier’s protocol using a MAO-Glo^TM^ Assay System (Promega, V1401). MAO-A (18 units/mL) or MAO-B (6 units/mL) (Sigma-Aldrich, Saint Louis, MO, USA, 25 µL/well), 160 µM (for MAO-A) or 16 µM (for MAO-B) (4*S*)-4,5-dihydro-2-(6-hydroxybenzothiazolyl)-4-thiazolecarboxylic acid (12.5 µL/well), a MAO substrate, and various concentrations of inhibitors (12.5 µL/well) were incubated at room temperature. Reactions were stopped after 60 min by adding reconstituted Luciferin Detection Reagent (50 µL/well). Then, 20 min after the addition of this reagent, the chemiluminescence of the wells was measured with a 2030 ARVO^TM^ X3 Multilabel Reader (PerkinElmer). For data processing, the same procedure as that for LSD1 inhibitory activity was used.

## 5. Conclusions

In summary, we described the design, synthesis, and in vitro evaluation of the LSD1 inhibitory activities of novel histone H3 peptide-based LSD1 inactivators **2a**–**c** incorporating α,α-disubstituted amino acids as γ-turn inducers. We revealed that the peptides **2a**–**c** were potent and selective LSD1 inactivators compared to reference peptide **1**. In particular, peptide **2b** incorporating one Acc on each side of lysine residue bearing a PCPA moiety was the most potent and selective LSD1 inactivator. These findings are useful for the further development of histone H3 peptide-based LSD1 inactivators. Detailed studies of peptides incorporating γ-turn inducers are underway in our laboratory.

## Figures and Tables

**Table 1:** LSD1 and MAO inhibitory activities of peptides **2a**–**c**.

Compound	IC_50_ (µM) ^1^		
	LSD1	MAO-A	MAO-B
PCPA	16.5 ± 1.9	6.00 ± 1.38	6.54 ± 0.51
**1**	0.126 ± 0.002	>10	>10
**2a**	0.0891 ± 0.0053	>10	>10
**2b**	0.0584 ± 0.0025	>10	>10
**2c**	0.0724 ± 0.0040	>10	>10

^1^ Values are means ± S.D. of at least three experiments.

## Supplementary Materials

Figure S1: Tine-dependent inhibition of LSD1 by **2a**, **2b** and **2c**, Figure S2: KDM4C inhibitory activities of **2a**, **2b** and **2b**, Figure S3: View of the binding pose of a model peptide based on **2b** in the active pocket of LSD1.
