# Supplementary material for: Design, Synthesis, and In Vitro Evaluation of Novel Histone H3 Peptide-Based LSD1 Inactivators Incorporating α,α-Disubstituted Amino Acids with γ-Turn-Inducing Structures
Source: Molecules. 2018 May 6;23(5):1099. doi: 10.3390/molecules23051099 (PMC6099693; doi:10.3390/molecules23051099)
Supplement: Supplementary file 1 [file molecules-23-01099-s001.pdf]

Supplementary information

Communication

Design, Synthesis, and In Vitro Evaluation of Novel Histone H3 Peptide-Based LSD1 Inactivators Incorporating α, α-Disubstituted Amino Acids with γ-Turn-Inducing Structures

Yosuke Ota [1,*], Taeko Kakizawa [2], Yukihiro Itoh [1] and Takayoshi Suzuki [1,3,*]

[1] Department of Chemistry, Graduate School of Medical Science, Kyoto Prefectural University of Medicine, 1-5 Shimogamohangi-Cho, Sakyo-Ku, Kyoto 606-0823, Japan; itohy@koto.kpu-m.ac.jp (Y.I.)
[2] Department of Chemistry and Biochemistry, School of Advanced Science and Engineering, Waseda University, Shinjuku, Tokyo 169-8555, Japan; w499487@aoni.waseda.jp (T.K.)
[3] CREST, Japan Science and Technology Agency (JST), 4-1-8 Honcho, Kawaguchi, Saitama 332-0012, Japan
* Correspondence: y-ota@koto.kpu-m.ac.jp (Y.O.); suzukit@koto.kpu-m.ac.jp (T.S.)

Table of contents

Supplementary Figure S1 Tine-dependent inhibition of LSD1 by **2a**, **2b** and **2c** ··························S2
Supplementary Figure S2 KDM4C inhibitory activities of **2a**, **2b** and **2b** ·······························S3
Supplementary Figure S3 View of the binding pose of a model peptide based on **2b** in the active pocket of LSD1 ··S4
Supplementary method ··S5
Supplementary reference ···S6

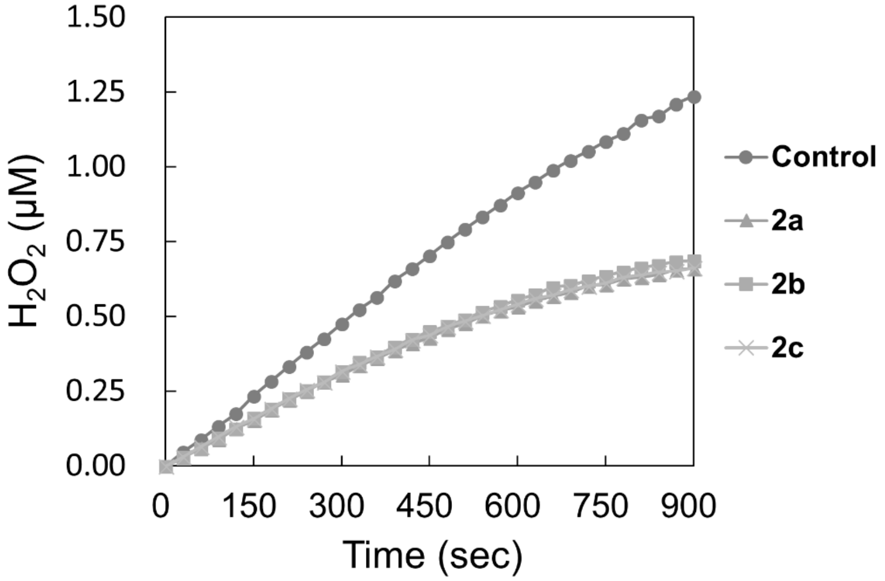

Supplementary Figure S1. Time-dependent inhibition of LSD1 by **2a**, **2b** and **2c** at a concentration of 0.2 µM.

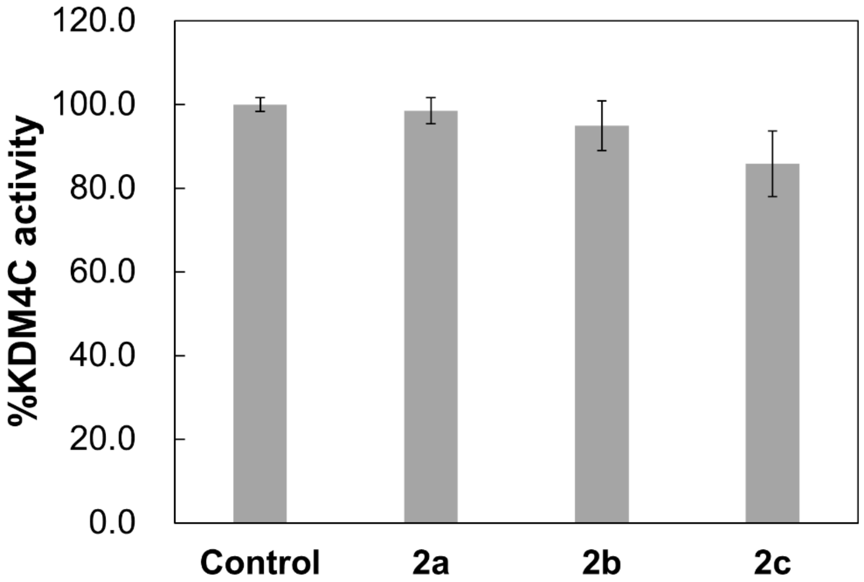

Supplementary Figure S2. KDM4C inhibitory activities of **2a**, **2b** and **2b** at a concentration of 10 μM.

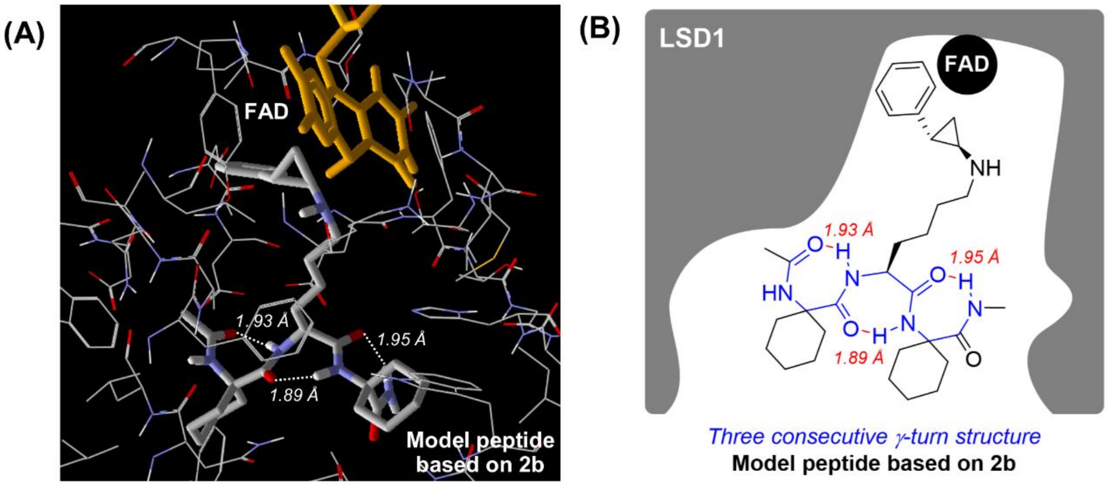

Supplementary Figure S3. View of the binding pose of the model peptide based on **2b** in the active pocket of LSD1. (A) View of the secondary structure of the model peptide in the active pocket of LSD1. Intramolecular hydrogen bonds are indicated in white and their lengths are shown in *italic*. (B) Schematic diagram of the secondary structure of the model peptide in the active pocket of LSD1. Intramolecular hydrogen bonds are indicated in red and their lengths are shown in *italic*.

Supplementary method

Kinetic LSD1 assay

The kinetic analysis for LSD1 was performed using a LSD1 fluorescent assay kit (Enzo life science, BML-AK544-0001).[1] A mixture of various concentrations of inhibitors (0.2 μM), CeLLestialTM Red, HRP, H3K4me2 peptide (20 μM), and LSD1 (0.5 μg/well) were deposited in all wells. Every 30 seconds, for a period of 15 minutes, the fluorescence of the wells was measured on a 2030 ARVO^{TM} X3 multilabel reader (PerkinElmer; excitation: 540 nm, detection: 590 nm). In order to determine the time-dependent inhibition of LSD1 by **2a-c**, reactions were initiated by addition of the enzyme to assay solutions containing the substrate and a fixed concentrations of inhibitors in a manner similar to that used in the enzyme inhibition assays described in main text.

AlphaLISA assay

Assays for KDM4 inhibitory activity were performed using alphaLISA assays.[2] A mixture of **2a-c** (10 μM) and KDM4C (10 nM) was pre-incubated in each well of 384-well plate with gently shaking (250 rpm) at room temperture for 15 minutes, and then each reaction was initiated by addition of a mixture of tri-methyl histone H3 (21-44) lysine 36 peptide with a biotin-tag (100 nM), α-ketoglutaric acid potassium salt (50 μM), ammonium iron(II) sulfate hexyahydrate (5 μM) and (+)-sodium ascorbate (100 μM) in assay buffer into all wells except blank wells. After 60 minutes reaction (room temperature, 250 rpm), a suspension of anti-di-methyl-histone H3 lysine 36 alphaLISA acceptor beads (0.5 μg/well) in 1x epigenetics buffer was added and the resulting mixtures were incubated for 60 minutes (room temperature; 250 rpm). Then, the reactions were incubated with alpha streptavidin donor beads (0.5 μg/well) in 1x epigenetics buffer for further 60 minutes. Finally, the alphaLISA signal of the wells was measured on a Ensight Reader (PerkinElmer) and % KDM4C activity was calculated from the signal readings of inhibitor wells relative to those of control wells.

Docking study

The initial structure of the model peptide [Ac-Acc-Lys (PCPA)-Acc-NHMe] for docking study was constructed using the Gaussian 09 package.[3] The structural optimization was carried out using the M06-2X variant of density functional theory (DFT),[4] which is often used for long distance interactions, with the 6-31G* basis set in the gas phase. The optimized structure was considered as minima in case of all harmonic frequencies being positive.

Docking and subsequent scoring were carried out using the Molegro Virtual Docker 6 software.[5] Coordinates of LSD1 completed with FAD-*N*-propargyl lysine peptide adduct were obtained from the Brookhaven Protein Data Bank (PDB code 2UXN). Hydrogen atoms were introduced computationally at appropriate positions, while water molecules, cofactors and *N*-propargyl lysine peptide were removed. FAD was converted to a cofactor, and the initial structure of the model peptide constructed by Gaussian 09

package was defined as a ligand. The structures of LSD1-bound model peptide were constructed by MolDock, which is based on a heuristic search algorithm that combines a differential evolution with a cavity prediction algorithm. Binding cavity predictions were based on the van der Waals force and one of the resulting cavities was chosen as a ligand-binding site. The following docking parameters were obtained: grid resolution, 0.30; max iterations, 1,500; population size, 50; energy threshold, 100.00; simplex evolution, 300 (max steps) and 1.00 (neighbour distance factor); search space, (X, Y, Z) = (65.64, 47.97, 35.60) with radius 10; distans constrains I (for N atom of cyclopropylamine of the model peptide), constratin center (X, Y, Z) = (64.51, 54.11, 34.28) with hard constrain between minimum 0 to maximum 2.0; distans constraints II (for α-C atom of lysine on the model peptide), constratint center (X, Y, Z) = (65.64, 47.97, 35.60) with hard constraint between minimum 0 to maximum 2.0.

Supplementary reference

1. Ota, Y.; Itoh, Y.; Kaise, A.; Ohta, K.; Endo, Y.; Masuda, M.; Sowa, Y.; Sakai, T.; Suzuki, T. Targeting Cancer with PCPA-Drug Conjugates: LSD1 Inhibition-Triggered Release of 4-Hydroxytamoxifen. *Angew. Chem. Int. Ed.* **2016**, *55*, 16115–16118, doi:10.1002/anie.201608711.
2. Normand, C.; Arcand, M.; Blouin, J.; Caron, M.; Labonté, A.; Beaudet, L.; Padrós, J. AlphaLISA JMJD2A Histone H3-Lysine 36 Demethylase Assay. Perkin Elmer, Inc. Technical Note: AlphaLISA#8.
3. Frisch, M. J.; Trucks, G. W.; Schlegel, H. B.; Scuseria, G. E.; Robb, M. A.; Cheeseman, J. R.; Scalmani, G.; Barone, V.; Petersson, G. A.; Nakatsuji, H.; Li, X.; Caricato, M.; Marenich, A. V.; Bloino, J.; Janesko, B. G.; Gomperts, R.; Mennucci, B.; Hratchian, H. P.; Ortiz, J. V.; Izmaylov, A. F.; Sonnenberg, J. L.; Williams-Young, D.; Ding, F.; Lipparini, F.; Egidi, F.; Goings, J.; Peng, B.; Petrone, A.; Henderson, T.; Ranasinghe, D.; Zakrzewski, V. G.; Gao, J.; Rega, N.; Zheng, G.; Liang, W.; Hada, M.; Ehara, M.; Toyota, K.; Fukuda, R.; Hasegawa, J.; Ishida, M.; Nakajima, T.; Honda, Y.; Kitao, O.; Nakai, H.; Vreven, T.; Throssell, K.; Montgomery, J. A., Jr.; Peralta, J. E.; Ogliaro, F.; Bearpark, M. J.; Heyd, J. J.; Brothers, E. N.; Kudin, K. N.; Staroverov, V. N.; Keith, T. A.; Kobayashi, R.; Normand, J.; Raghavachari, K.; Rendell, A. P.; Burant, J. C.; Iyengar, S. S.; Tomasi, J.; Cossi, M.; Millam, J. M.; Klene, M.; Adamo, C.; Cammi, R.; Ochterski, J. W.; Martin, R. L.; Morokuma, K.; Farkas, O.; Foresman, J. B.; Fox, D. J., Gaussian, Inc., Wallingford CT **2009**.
4. Zhao, Y., Schultz, N. E. & Truhlar, D. G. J. Design of density functionals by combining the method of constraint satisfaction with parametrization for thermochemistry, thermochemical kinetics, and noncovalent interactions. *J. Chem. Theory. Comput.* **2006**, *2*, 364–382, doi: 10.1021/ct0502763.
5. Itoh, Y.; Ogasawara, D.; Ota, Y.; Mizukami, T.; Suzuki, T. Synthesis, LSD1 Inhibitory Activity, and LSD1 Binding Model of Optically Pure Lysine-PCPA Conjugates. *Comput. Struct. Biotechnol. J.* **2014**, *9*, e201402002, doi:10.5936/csbj.201402002.
